# Increasing Predominance of Norovirus GII.17 over GII.4, United States, 2022–2025

**DOI:** 10.3201/eid3107.250524

**Published:** 2025-07

**Authors:** Leslie Barclay, Jan Vinjé

**Affiliations:** Centers for Disease Control and Prevention, Atlanta, Georgia, USA

**Keywords:** norovirus, GII.17, GII.4, viruses, enteric infections, United States

## Abstract

Norovirus GII.17 outbreaks in the United States increased from <10% during the 2022–23 season to 75% during the 2024–25 season, surpassing the number of GII.4 outbreaks. The norovirus season also started earlier in 2024–25 than in previous seasons. Continued norovirus surveillance is needed to detect and monitor emerging strains.

Norovirus is the leading cause of acute gastroenteritis outbreaks in the United States (*1*). Genetically, noroviruses are classified into 10 genogroups (GI–GX) and further into 48 genotypes and 60 P-types (*2*). Most outbreaks are caused by genogroup GI and GII viruses. During 2011–2024, GII.4 viruses have caused >50% of US outbreaks each season (defined as September 1 of one year through August 31 of the next) (*3*).

Laboratory surveillance of norovirus in the United States is conducted through CaliciNet, a network of public health laboratories from local, state, and federal agencies (*4*). As previously reported (*5*), several countries, including the United States, observed an increase in GII.17 cases and outbreaks during the 2023–24 season. Initially, GII.17 outbreaks in the United States remained below GII.4 outbreak numbers. We present an updated analysis on the increase of GII.17 outbreaks since September 2022.

We analyzed the genotype distribution of outbreaks uploaded to CaliciNet during September 2022–April 2025. We grouped genotypes into 3 categories: GII.17, GII.4 (including GII.4 Sydney, GII.4 San Francisco, and GII.4 Wichita), and other genotypes (all other GI and GII genotypes). Complete GII.17 genome sequences for 2022–2024 have been reported previously (*5*), and strains representing the 2024–25 season are available from GenBank (accession nos. PV588655–8 and PV588796–7).

During the 2022–23 season, GII.17 accounted for 7.5% and GII.4 for 48.9% of all outbreaks (Figure 1). The next season (2023–24), the percentage of GII.17 outbreaks increased to 34.3%, whereas GII.4 outbreaks declined to 27.7% (Figure 1). By the 2024–25 season, GII.17 outbreaks had increased markedly to 75.4%, whereas GII.4 outbreaks further decreased to 10.7% (Figure 1). In addition, during 2022–23 and 2023–24, seasonality was primarily driven by GII.4 viruses, showing peak activity in February 2023 and March 2024, whereas during the 2024–25 season, norovirus peaked in January 2025 (Figure 2). 

**Figure 1:**
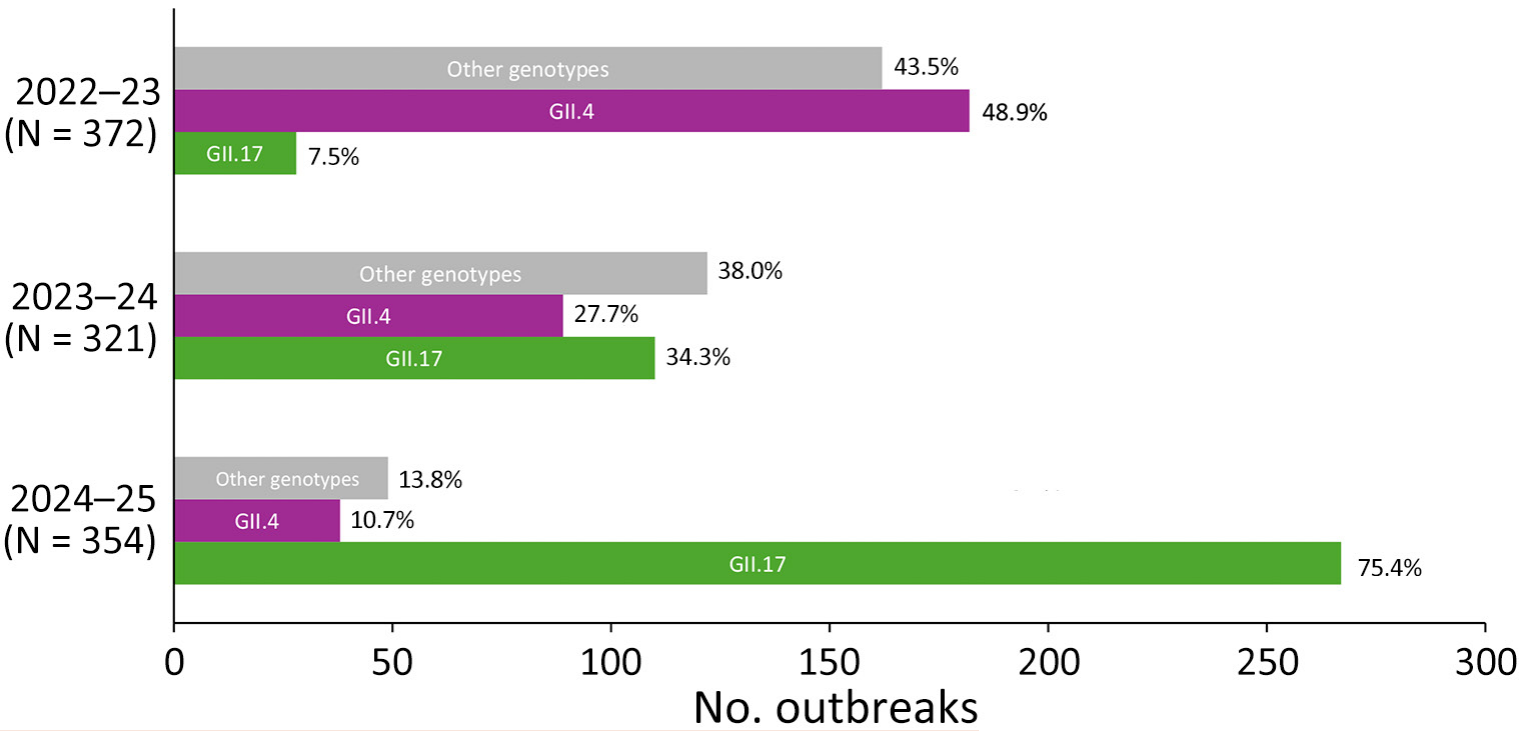
Increase in outbreaks of norovirus GII.17 over GII.4, United States, 2022–2025. Seasons are defined as September 1 of one year to August 31 of the next. The 2024–25 season is truncated to September 2024–April 2025. Other genotypes: GI.1, GI.2, GI.3, GI.4, GI.5, GI.6, GI.7, GII.1, GII.2, GII.3, GII.6, GII.7, GII.8, GII.10, GII.12, GII.13, GII.14, GII.21, GII.27, and GIX.1.

**Figure 2:**
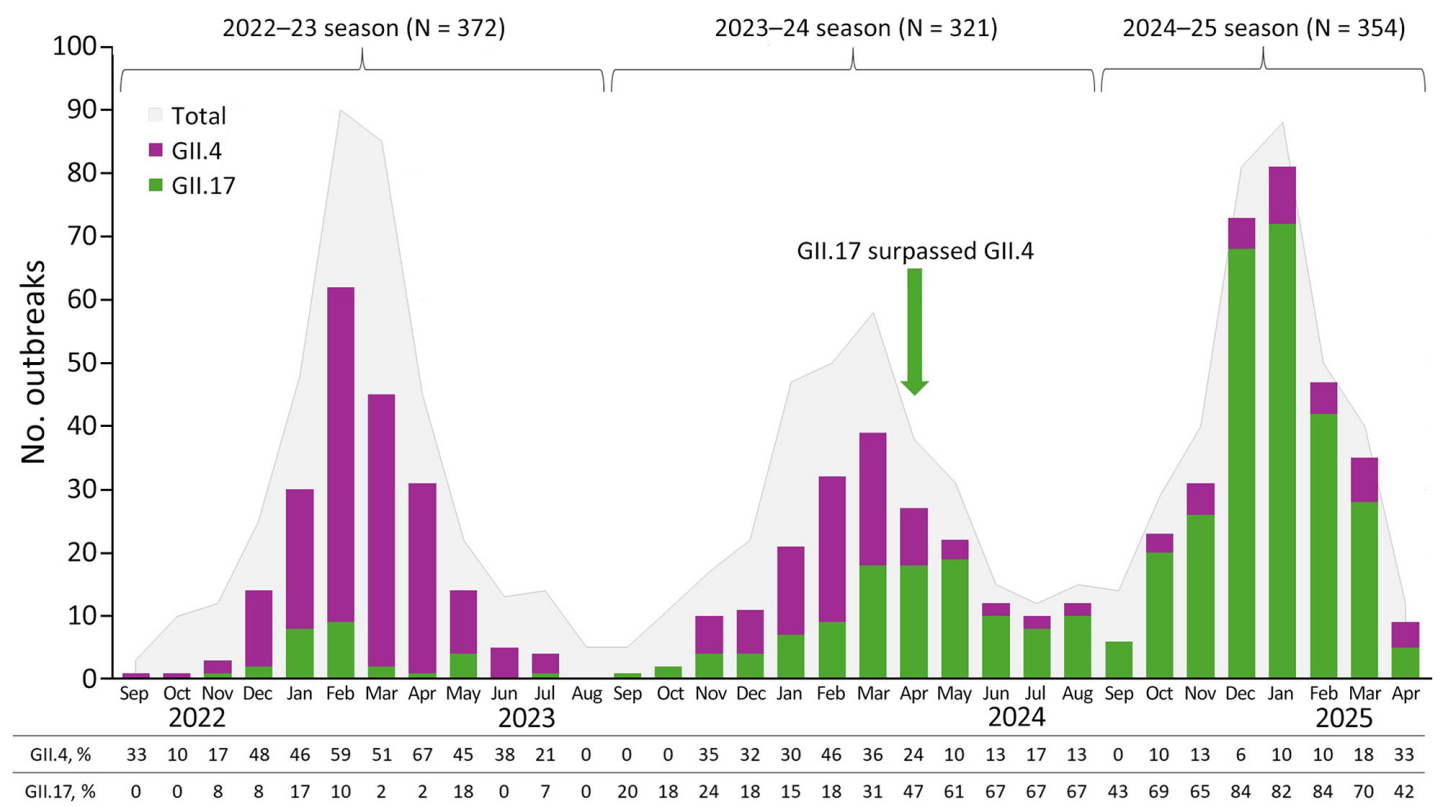
Numbers and percentages of norovirus GII.17 and GII.4 outbreaks compared with total number of outbreaks by month, United States, September 2022–April 2025. Seasons are defined as September 1 of one year to August 31 of the next. The 2024–25 season is truncated to September 2024–April 2025.

In April 2024, the percentage of GII.17 outbreaks increased to 47.4%, overtaking GII.4 outbreaks (23.7%), and from May 2024 through March 2025, GII.17 accounted for >50% of all outbreaks each month (Figure 2). During September–December of the 2024–25 season, GII.17 accounted for 46.3% of all outbreaks, compared with 13.4% in 2022–23 and 17.1% in 2023–24 during the same 4-month period. We observed no regional differences in distribution of GII.4 and GII.17 outbreaks.

Our data highlight a substantial shift in genotype distribution of norovirus outbreaks in the United States from 2022 to 2025, with GII.17 emerging as the predominant genotype. That shift coincides with a notable decline in GII.4, which has traditionally been the leading cause of US outbreaks. In 2014, several countries in Asia reported a GII.17 strain that completely replaced GII.4 Sydney (*6*). That strain was also detected in several countries in Europe and the United States (*6*–*8*), but in 2016, GII.4 viruses rebounded across the globe and became predominant again (*4*,*9*). The likely ancestor of the current GII.17 virus is a strain that caused an outbreak in Romania in 2021 (*5*).

In the United States, the norovirus season typically starts in early December (*1*,*4*), but in 2024–25, the onset of the season was in early October 2024, an observation further supported by data from NoroSTAT (https://www.cdc.gov/norovirus/php/reporting/norostat-data.html). In 2023–24, we observed a prolonged outbreak season with cases continuing into the summer, likely the result of sustained circulation of GII.17. In contrast, the 2022–23 and 2024–25 seasons showed a more typical pattern, in which cases rapidly decline during the spring months. Cannon et al. (*4*) reported a decline after the winter peak in all 3 seasons they studied, and Wikswo et al. (*1*) reported a yearly decrease in the number of outbreaks each March over a 10-year study period. Those and other studies have also shown that GII.4 viruses are the main driver for norovirus seasonality (*1*,*4*,*7*). With the decrease of GII.4 outbreaks since 2024, it is too soon to determine whether GII.17 viruses will continue to cause an earlier onset of the norovirus season.

Our data highlight the value of CaliciNet, a national laboratory-based surveillance network that uses standardized norovirus typing methods, including whole-genome sequencing. Norovirus surveillance plays a crucial role in detecting and monitoring emerging strains, serving as an early warning system that enables rapid response to outbreak investigations and timely implementation of interventions and prevention strategies.

In conclusion, GII.17 has caused 75% of all norovirus outbreaks during the 2024–25 season so far, thereby replacing GII.4 as the predominant norovirus outbreak strain in the United States. Additional sequence analysis of complete GII.17 genomes and identification of cross-protective neutralizing antibodies of GII.17 compared with GII.4 viruses could help clarify whether GII.17 viruses will persist. Continued surveillance is needed to determine if this genotype remains the dominant genotype, as well as whether the norovirus season continues to start earlier than previous years. 
